# Wallenberg Syndrome After Leg Day Training: A Case Report

**DOI:** 10.7759/cureus.95084

**Published:** 2025-10-21

**Authors:** André Pereira, Cristina Silva, Filipa Gonçalves, Sara Freitas, Glória Alves

**Affiliations:** 1 Internal Medicine, Unidade Local de Saúde Alto Ave, Guimarães, PRT

**Keywords:** cervical artery dissection, ischemic stroke, posterior circulation stroke, vertebral artery dissection, wallenberg syndrome

## Abstract

Cervical artery dissection (CAD) is an important cause of ischemic stroke in young adults, often triggered by mild cervical trauma such as abrupt neck movements during physical exertion. Vertebral artery dissection can lead to posterior circulation strokes, including lateral medullary (Wallenberg) syndrome, presenting with characteristic neurological deficits.

We report the case of a 40-year-old male who developed right-sided occipital headache, vomiting, dysphagia, dysphonia, dizziness, and sensory disturbances shortly after isotonic lower-limb exercise. Clinical examination revealed signs consistent with right lateral medullary syndrome. Laboratory analysis revealed markedly elevated creatine phosphokinase, consistent with exertional rhabdomyolysis. Imaging studies demonstrated dissection and occlusion of the distal cervical right vertebral artery and acute infarction in the right lateral medulla. Genetic testing for connective tissue disorders was negative. The patient was managed with dual antiplatelet therapy, statins, and supportive care, including rehabilitation for bulbar symptoms. At discharge, he was clinically stable with partial improvement and referred for intensive neurorehabilitation.

This case highlights the importance of considering CAD in young patients with posterior circulation stroke symptoms after physical exertion. Early diagnosis using advanced imaging and prompt antithrombotic therapy is critical. Multidisciplinary management, including rehabilitation, is essential for optimizing recovery. Awareness of this entity may improve patient outcomes and reduce long-term disability.

## Introduction

Cervical artery dissection (CAD) refers to a tear in the wall of one of the neck arteries, most commonly the carotid or vertebral arteries. This process allows blood to enter the vessel wall, forming an intramural hematoma that can lead to luminal narrowing, occlusion, or distal embolization [1,2]. It represents a significant cause of ischemic stroke in young and middle-aged adults, accounting for approximately 15% to 25% of ischemic strokes in individuals under 50 years [3].

Among cervical artery dissections, vertebral artery dissection involves injury to the artery supplying the posterior circulation of the brain. When blood flow is compromised, infarction of the lateral medulla can occur, resulting in lateral medullary (Wallenberg) syndrome. This condition typically presents with ipsilateral facial sensory loss, contralateral body pain/temperature deficits, ataxia, dysphagia, hoarseness, nystagmus, vertigo, and Horner’s syndrome [4,5].

Although cervical artery dissection may be associated with trauma or connective tissue disorders, it can also arise spontaneously or following seemingly benign physical activity. Growing evidence suggests that abrupt neck movements, mechanical strain, or transient vascular shear stress during exertion can precipitate arterial dissection even in individuals without identifiable risk factors [1,6].

This case report describes a young, physically active male who developed vertebral artery dissection and subsequent Wallenberg syndrome shortly after performing lower-limb resistance training (“leg day”). It illustrates a rare but clinically important association between intense exercise and posterior circulation stroke. By presenting this case, we aim to raise awareness among clinicians about exertion-related vascular injury and to emphasize the importance of early recognition and imaging in patients presenting with posterior circulation symptoms after physical activity.

## Case presentation

A 40-year-old previously healthy male presented to the Emergency Department with a sudden onset of right occipital headache, vomiting, dysphagia, dysphonia, dizziness, and sensory disturbances starting 30 minutes after isotonic leg training. The patient reported performing a high-intensity workout consisting primarily of Weighted squats and leg presses. During these exercises, he maintained repeated episodes of neck extension and mild rotation while exerting strain, particularly when stabilizing posture under load. He denied any direct neck trauma or recent infection.

Neurological exam on admission revealed signs consistent with a right lateral medullary syndrome (Wallenberg’s syndrome), including right-sided ptosis, anisocoria (incomplete Horner’s syndrome), severe dysphagia requiring nasogastric feeding, dysphonia, right hemifacial hypoesthesia, and contralateral impairment of pain and temperature sensation. These neurological deficits correspond to involvement of the nucleus ambiguus, spinal trigeminal nucleus, and spinothalamic tract, explaining the patient’s dysphagia, dysphonia, ipsilateral facial sensory loss, and contralateral body pain and temperature deficits. The partial Horner’s syndrome reflects disruption of descending sympathetic pathways within the lateral medulla.

Laboratory tests revealed a marked elevation of creatine phosphokinase (4387 U/L), consistent with exertional rhabdomyolysis, with preserved renal function and normal electrolyte levels (Table 1).

**Table 1:** Blood test results performed at hospital admission

Test	Result	Reference range
Creatine phosphokinase (UI/L)	4387	34 - 171
Urea (mg/dL)	46	15 - 39
Creatinine (mg/dL)	0.82	0.70 - 1.30
Sodium (mEq/L)	142	135 – 136
Potassium (mEq/L)	3.52	3.5 - 5.1
Chloride (mEq/L)	108	95 - 105
Calcium (mg/dL)	10.0	8.3 - 10.6
Phosphorus (mg/dL)	3.8	2.5 - 4.9
Magnesium (mg/dL)	2.28	1.6 - 2.6

**Figure 1:**
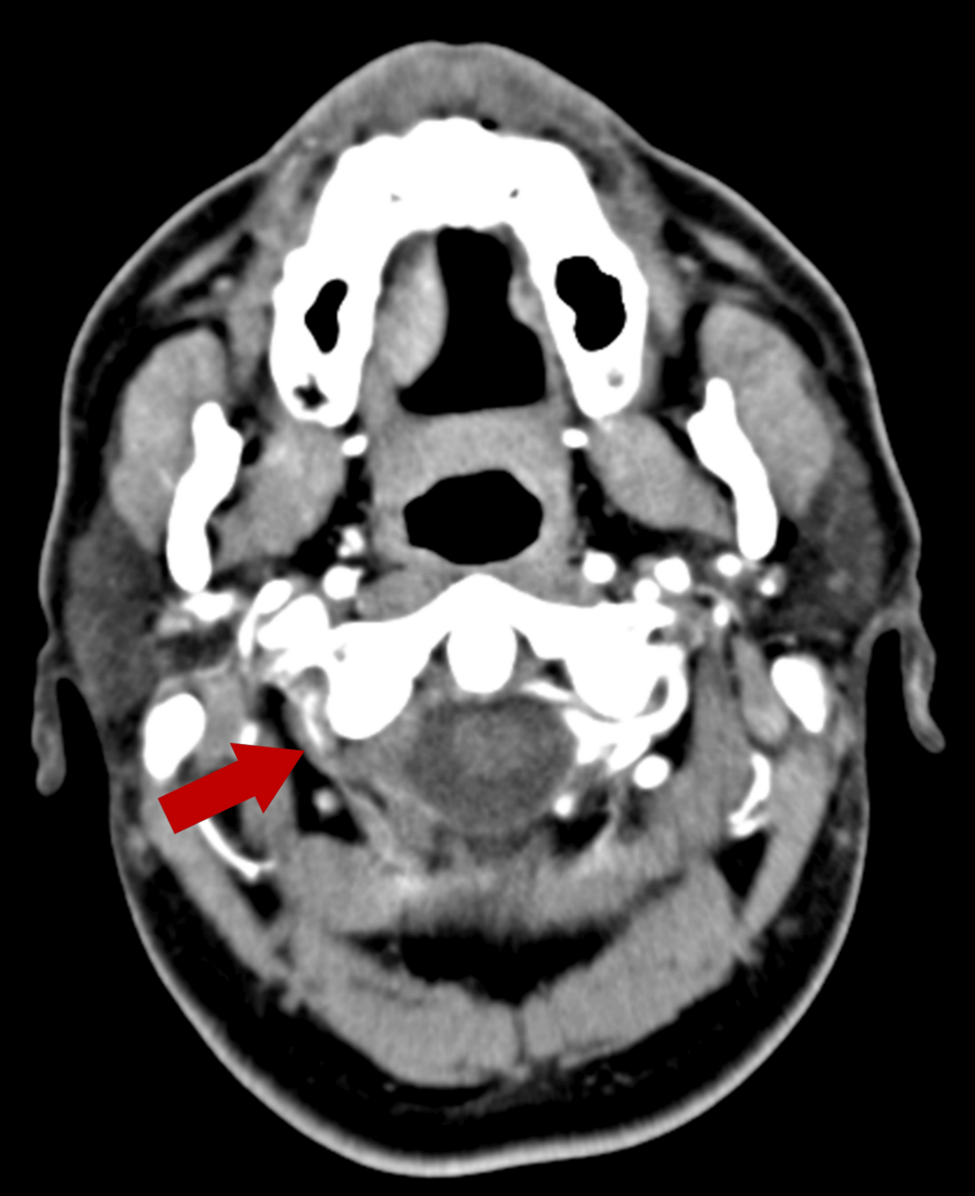
CT angiography scan CT angiography demonstrates tapering and focal occlusion of the distal cervical segment (V3) of the right vertebral artery (red arrow), consistent with arterial dissection. The absence of contrast opacification beyond this point supports a diagnosis of flow-limiting intramural hematoma. This finding correlates with the patient’s posterior circulation ischemic symptoms and localizes the vascular injury site.

Computed tomography angiography demonstrated tapering and occlusion of the distal cervical segment of the right vertebral artery, consistent with arterial dissection (Figure 1).

The patient was admitted to the Stroke Unit. The initial management strategy comprised dual antiplatelet therapy (aspirin and clopidogrel), high-intensity statin therapy, close neurological monitoring, intravenous fluid therapy for rhabdomyolysis, and early initiation of physical rehabilitation. 

The patient’s condition remained generally stable, with gradual improvement in some deficits, though he persisted with significant dysphagia (requiring enteral nutrition), dysphonia, and sensory disturbances. He experienced recurrent hiccups, attributed to the bulbar lesion, managed with gabapentin.

**Figure 2:**
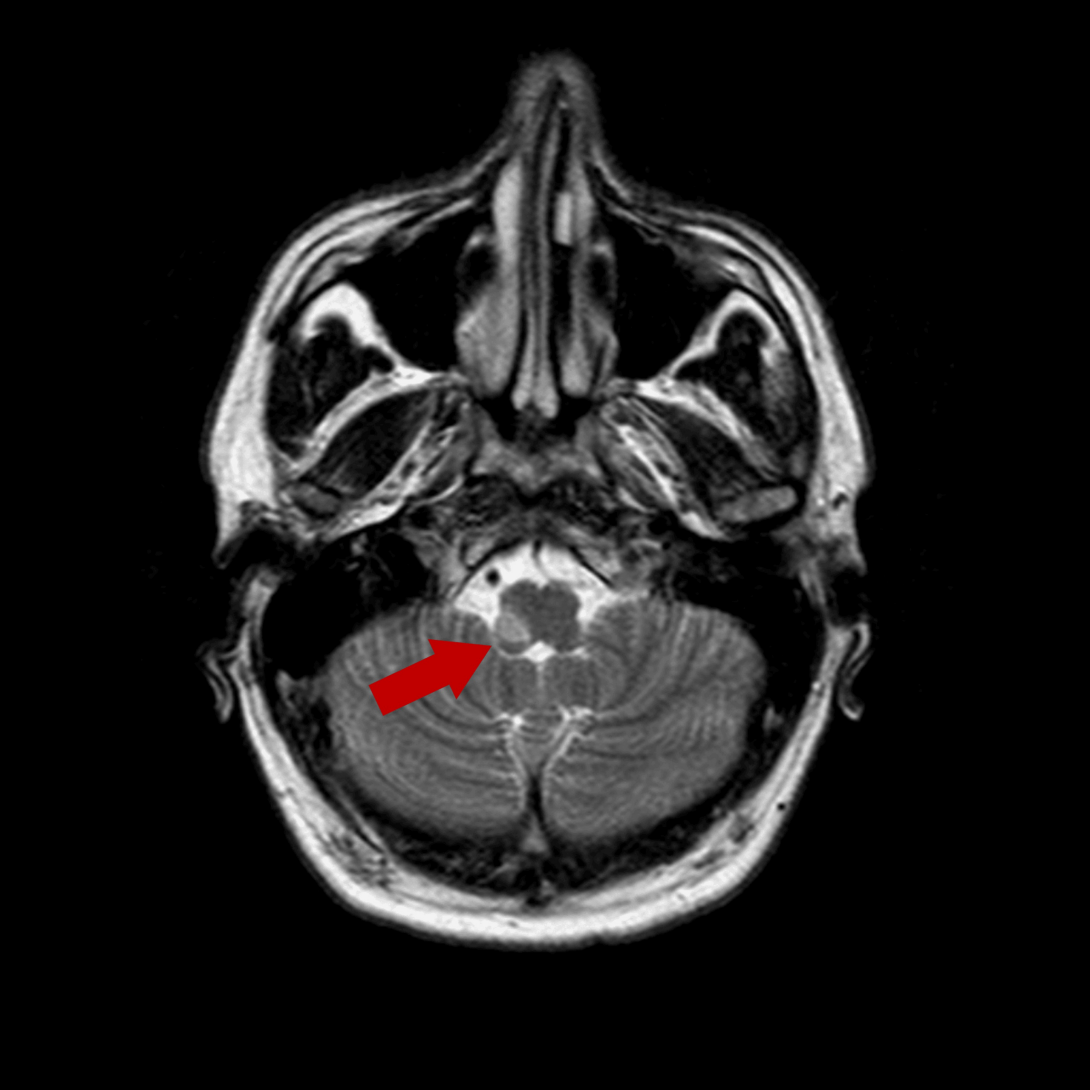
Brain MRI Axial diffusion-weighted MRI reveals a hyperintense lesion involving the right lateral medulla (red arrow), corresponding to restricted water diffusion consistent with acute infarction in the territory of the right vertebral and posterior inferior cerebellar arteries. This radiological pattern is characteristic of lateral medullary (Wallenberg) syndrome and explains the patient’s dysphagia, dysphonia, and contralateral sensory deficits.

**Figure 3:**
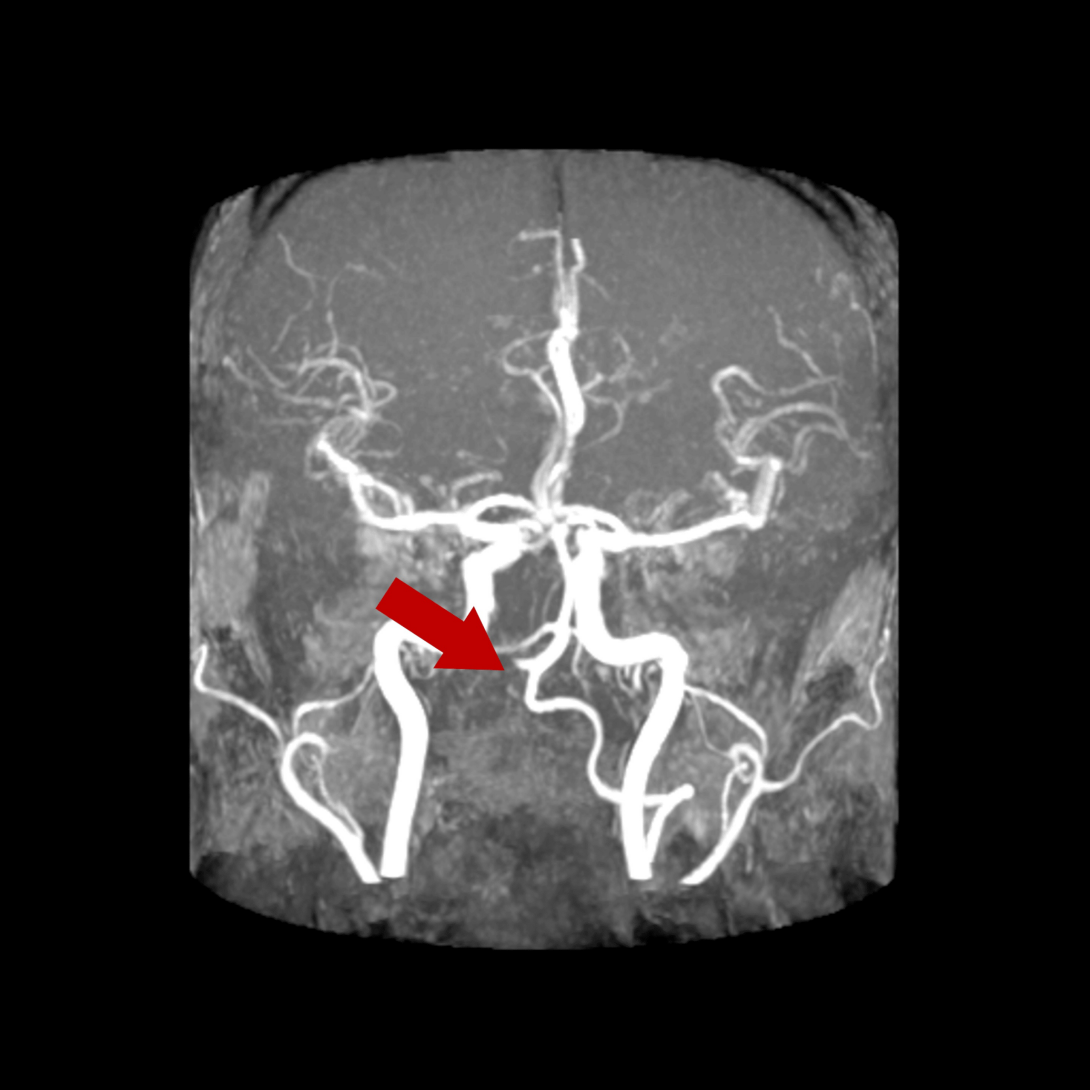
3D-TOF magnetic resonance angiography (MRA) 3D-TOF MRA shows the absence of an intracranial flow signal within the right vertebral artery (red arrow), confirming loss of patency distal to the dissection site. The finding complements the CT angiography results and illustrates the hemodynamic consequence of vertebral artery dissection leading to medullary infarction. TOF: time of flight

Five days after admission, brain magnetic resonance imaging (MRI) with angiographic study using the 3D time-of-flight (TOF) technique confirmed an acute infarction involving the right lateral medulla in the territory of the right vertebral/posterior inferior cerebellar artery (PICA) (Figure 2), with absent intracranial flow signal in the right vertebral artery (Figure 3). These imaging findings correlated precisely with the patient’s neurological deficits and confirmed the diagnosis of vertebral artery dissection leading to Wallenberg syndrome.

From the remaining etiological workup, serologies for Human Immunodeficiency Virus, hepatitis B and C, and syphilis were negative, as was autoimmune screening. Transesophageal echocardiography ruled out a patent *foramen ovale*, and electroencephalography showed a normal alpha rhythm without focal abnormalities.

Screening for mutations in the ABCC6, COL3A1, COL4A1, COL4A3, COL4A4, COL5A1, COL5A2, FBN1, MEF2A, and RNF213 genes yielded negative results.

Throughout hospitalization, he remained hemodynamically stable with no significant neurological deterioration.

The final diagnosis included lateral medullary syndrome (Wallenberg syndrome) secondary to spontaneous dissection of the right vertebral artery (V3 segment), ischemic stroke of the posterior circulation, and exertional rhabdomyolysis. Management included dual antiplatelet therapy for three months.

At discharge, the patient was referred to intensive rehabilitation therapy, with a recommendation to avoid air travel for the following month.

During follow-up, he remained clinically stable without recurrence of ischemic events.

## Discussion

CAD represents a significant cause of ischemic stroke in young and middle-aged adults. Both carotid and vertebral arteries can be affected; however, vertebral artery dissections are particularly associated with posterior circulation strokes, including Wallenberg syndrome, as seen in our patient [5].

Exercise-induced CAD is an increasingly recognized phenomenon, with even mild cervical trauma or abrupt neck movements during high-intensity workouts serving as potential triggers [1,4,6]. In our patient, the ischemic event followed an isotonic lower-limb training session, which likely involved repetitive neck extension and rotation, contributing to vertebral artery injury. Although exertional dissections are well documented, reports specifically linking lower-limb exercise without direct neck trauma to vertebral artery dissection are rare, making this case an uncommon and noteworthy presentation. Similar mechanisms have been described in exercise-induced carotid dissections, underscoring the need for vigilance in younger, physically active patients presenting with stroke-like symptoms [6].

The marked creatine phosphokinase (CPK) elevation observed in this patient most likely represents exertional rhabdomyolysis that developed several hours after intense lower-limb exercise, consistent with the delayed enzymatic rise typically seen following vigorous physical activity [6]. The combination of heavy-weighted squats and sustained neck stabilization under load may have contributed simultaneously to muscular strain and vertebral artery stress.

The pathophysiological link between lower-limb exertion and cervical artery dissection likely results from combined hemodynamic and biomechanical stress. Transient surges in blood and intrathoracic pressure during resistance exercises such as squats and leg presses can increase arterial wall tension and predispose to intimal injury [1,6]. Repetitive neck extension and rotation used to stabilize posture under heavy load may further enhance shear stress on the vertebral artery [4,5]. Similar non-traumatic dissections have been reported after weight training, yoga, and other sports, supporting the role of minor repetitive biomechanical forces in precipitating arterial injury [1,6,7].

The clinical presentation of vertebral artery dissection can be heterogeneous, ranging from isolated headache or neck pain to severe neurological deficits. Lateral medullary syndrome is characterized by ipsilateral facial hypoesthesia, contralateral body pain and temperature deficits, dysphagia, dysphonia, ataxia, vertigo, and partial Horner’s syndrome [5]. Early recognition of these characteristic findings is crucial, as delays can result in progression of infarction and increased morbidity [4,5]. In our case, the combination of occipital headache, vomiting, dysphagia, dysphonia, and sensory disturbances prompted urgent imaging, facilitating timely diagnosis.

Advanced imaging is central to confirming CAD and assessing its extent. CT angiography remains a rapid and accessible modality for detecting arterial tapering, occlusion, or pseudoaneurysm formation [2]. Complementary Brain 3D-TOF MRA enhances visualization of the intramural hematoma and infarcted territory, particularly in the posterior circulation [8]. In our patient, CT angiography revealed distal right vertebral artery occlusion, while MRA confirmed acute infarction in the right lateral medulla and absent flow in the corresponding vertebral artery segment. This imaging strategy is consistent with current recommendations for suspected CAD and posterior circulation stroke [4,8].

Management of CAD is primarily antithrombotic, aimed at preventing stroke progression or recurrence. Evidence from the CADISS trial indicates no significant difference between anticoagulation and antiplatelet therapy for stroke prevention in stable patients, supporting individualized treatment decisions [9]. In practice, dual antiplatelet therapy (aspirin plus clopidogrel) represents a reasonable approach for patients without contraindications, as applied in this case [10]. Concurrent statin therapy may provide additional vascular protection and is recommended in ischemic stroke management [11].

Supportive care addressing secondary complications such as rhabdomyolysis in our patient and management of bulbar symptoms, including hiccups, is integral to optimizing functional recovery [5,10].

Etiological evaluation should also consider underlying connective tissue disorders or arteriopathies, including Ehlers-Danlos, Marfan, and RNF213-related syndromes [6]. In our patient, extensive genetic testing was negative, suggesting a mechanical etiology related to exertional neck strain rather than hereditary predisposition. This underscores the multifactorial nature of CAD, in which environmental and anatomical factors interact to precipitate arterial injury [1,6].

Prognosis following posterior circulation dissection is generally favorable when promptly recognized and managed, with most patients achieving good functional recovery and a low recurrence rate under appropriate antithrombotic therapy [9,10]. Nevertheless, residual bulbar deficits such as dysphagia and dysphonia may persist, significantly affecting quality of life and nutritional status [5,10]. Early referral to intensive rehabilitation and multidisciplinary follow-up is therefore essential to maximize recovery and functional outcomes [5]. Comprehensive secondary prevention, including modification of exertional activities and temporary avoidance of factors that may increase arterial stress, such as air travel during the acute phase, is also recommended to minimize recurrence risk and promote vascular healing [5,6,10].

## Conclusions

This case reinforces the need for heightened awareness of CAD as an important cause of posterior circulation stroke, particularly in younger and physically active individuals following intense exertion. The patient’s presentation, together with characteristic imaging findings of a right lateral medullary infarct secondary to vertebral artery dissection, illustrates the classical clinical and radiological features of this condition. Clinicians should maintain a high index of suspicion for vertebral artery dissection in similar contexts, even in the absence of direct neck trauma or conventional vascular risk factors.

Early use of vascular imaging, including CT angiography or MR angiography, is crucial for prompt diagnosis and initiation of appropriate antithrombotic therapy. When recognized and treated promptly, vertebral artery dissections are associated with favorable neurological outcomes and excellent recovery potential. Preventive and rehabilitative considerations are equally important for active individuals, including education about early posterior circulation warning symptoms, appropriate exercise modification, and gradual, supervised return to physical activity. Comprehensive multidisciplinary management that integrates acute care, rehabilitation, risk factor control, and patient education remains essential to optimize recovery, reduce recurrence, and support long-term functional independence.
